# NCOA4 initiates ferritinophagy by avidly binding GATE16 using two short linear interaction motifs

**DOI:** 10.26508/lsa.202503611

**Published:** 2026-02-04

**Authors:** April Lee, Joseph H Davis

**Affiliations:** 1 Department of Biology, Graduate Program, Massachusetts Institute of Technology , Cambridge, MA, USA; 2 Computational and Systems Biology, Graduate Program, Massachusetts Institute of Technology , Cambridge, MA, USA

## Abstract

Cells carefully regulate cytosolic iron, which is a vital enzymatic cofactor, yet is toxic in excess. In mammalian cells, surplus iron is sequestered in ferritin cages that, in iron-limiting conditions, can be degraded through the selective autophagy pathway ferritinophagy to liberate free iron. Prior work identified the ferritinophagy receptor protein NCOA4, which links ferritin and LC3/GABARAP-family member GATE16, effectively tethering ferritin to the autophagic machinery. Here, we elucidate the molecular mechanism underlying this interaction, discovering two short linear motifs in NCOA4 that each bind GATE16 with weak affinity. These binding motifs are highly avid and, in concert, support high-affinity NCOA4•GATE16 complex formation. We further find the minimal NCOA4^383−522^ fragment bearing these motifs is sufficient for ferritinophagy and that both motifs are necessary for this activity. This work suggests a general mechanism wherein selective autophagy receptors can distinguish between the inactive pools of monomeric LC3/GABARAPs and the active oligomerized forms that drive autophagy. Finally, we find that iron decreases affinity of the NCOA4^383−522^ fragment for GATE16, providing a plausible mechanism for iron-dependent regulation of ferritinophagy.

## Abstract (precis)

This study shows that NCOA4 engages GATE16 through two short linear motifs that bind avidly to support ferritinophagy, revealing how multivalent LC3/GABARAP interactions confer specificity and suggesting that iron directly regulates NCOA4•GATE16 binding by occluding one motif.

## Introduction

All cells must tightly regulate nutrient levels, including metals such as iron that act as crucial cofactors for essential enzymes. Whereas ample labile iron is vital for cellular function, excess iron can be toxic due, in part, to its ability to catalyze the formation of damaging reactive oxygen species (Wang & Pantopoulos, 2011). Indeed, iron dysregulation has been connected to a variety of neurological diseases including hereditary ferritinopathy, and Alzheimer’s (Ward et al, 2014) and Parkinson’s (Sofic et al, 1988) diseases. Mammalian cells are thought to maintain an appropriate pool of cytosolic iron by balancing iron uptake and storage in ferritin cages with the release of ferritin-sequestered iron through degradation of these proteinaceous cages (Arosio et al, 2009).

One mechanism by which ferritin is degraded upon depletion of free iron pools is ferritinophagy, a type of selective autophagy (Mancias et al, 2015) wherein select cellular components are recognized by an autophagy receptor protein and encapsulated in a double-membraned vesicle that is targeted to the lysosome for proteolysis (Zaffagnini & Martens, 2016). Ferritinophagy is thought to rely on the protein nuclear receptor coactivator 4 (NCOA4), which was first identified as a selective autophagy receptor in an LC-MS/MS-based proteomics approach targeting proteins that copurified with autophagosomes (Mancias et al, 2014). The activity of NCOA4 is regulated through iron-dependent changes in affinity for ferritin (Zhao et al, 2024), as well as the E3 ligase HERC2, which can promote NCOA4 degradation via the proteasome (Mancias et al, 2015). An NCOA4 fragment composed of residues 383–522 (NCOA4^383−522^, hereafter) was subsequently found to be sufficient to bind directly to ferritin (Mancias et al, 2015; Hoelzgen et al, 2024).

To link the intended substrate and the autophagic machinery, selective cargo receptors typically bind to one or more of the six members of the microtubule-associated proteins 1A/1B light chain 3 (LC3/GABARAP) family of integral autophagosomal proteins (Lee & Lee, 2016). These homologs, which include LC3A, LC3B, LC3C, GABARAP, GEC1, and GATE16, exchange between a monomeric soluble form and a lipid-conjugated form wherein they are covalently linked to phosphatidylethanolamine headgroups of phospholipids in the autophagosomal membrane (Kabeya et al, 2004; Kraft et al, 2014). Selective autophagy receptors are thought to tether bound substrates to the autophagosomal membrane by simultaneously binding to LC3/GABARAPs via a short linear motif known as an LC3-interacting region (LIR). These motifs are hydrophobic in nature and have a consensus sequence of [W/F/Y]-X-X-[I/L/V], often accompanied by an N-terminal patch of acidic residues (Johansen & Lamark, 2020). Structural studies have further shown that the LC3/GABARAPs contain a LIR docking site (LDS) consisting of two hydrophobic pockets that support docking of the aromatic and aliphatic residues of the LIR motif (Johansen & Lamark, 2020).

Autophagy receptors are typically thought to associate with LC3/GAPARAPs using a single LIR, with affinity often in the low micromolar range (Johansen & Lamark, 2020). However, the LIR motif is common in the proteome and multiple instances can be found in most autophagy receptors, raising the possibility of highly avid, multivalent interactions with arrays of oligomerized or otherwise highly concentrated LC3/GABARAP-family proteins. In such a model, multiple binding motifs on the same receptor, each with weak intrinsic affinity, would act in concert to increase the effective receptor protein concentration and thus support high-affinity binding to multimerized LC3/GABARAPs (Lluís Garcés et al, 2009; Errington et al, 2019) such as those found on an autophagosomal membrane densely decorated by LC3/GABARAPs or tethered in proximity by other scaffolding proteins.

Here, to biochemically define the interactions supporting ferritinophagy, we extend the work of Mancias et al, who previously identified the LC3/GABARAP-family protein GATE16 as a strong NCOA4 interactor (Mancias et al, 2014). Specifically, using a purified NCOA4^383−522^ fragment, we identify and characterize two LIR-like motifs in NCOA4^383−522^ that each weakly bind to the LDS of GATE16. We further show that these LIR-like motifs are highly avid and that robust GATE16•NCOA4 complex formation requires oligomerized GATE16. Furthermore, we show that this minimal NCOA4^383−522^ fragment is sufficient for lysosomal degradation of ferritin in cells and that such degradation requires the two identified motifs. Finally, we demonstrate that binding of iron to NCOA4^383−522^ decreases its affinity for GATE16.

## Results

### NCOA4 binds directly to GATE16

**Figure 1.**
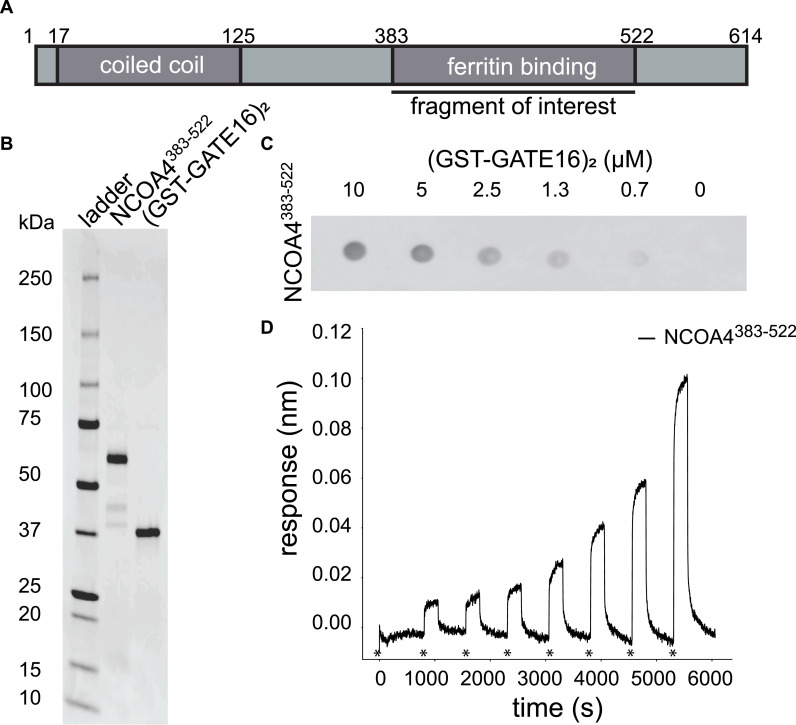
NCOA4^383−522^ binds directly to (GST-GATE16)_2_. **(A)** Schematic of NCOA4 domain architecture. The fragment of interest (NCOA4^383−522^) used in this study is noted. **(B)** SDS–PAGE gel of purified biotinylated AviTag-His_6_-NCOA4^383−522^-MBP (NCOA4^383−522^) and (GST-GATE16)_2_. **(C)** Far-Western blot of NCOA4^383−522^ binding to decreasing concentrations of (GST-GATE16)_2_, which was spotted on the membrane at the indicated concentration. Signal was detected by HRP-conjugated streptavidin directed to Avi-tagged NCOA4^383−522^, which was incubated on the membrane at a concentration of 0.1 μM. **(D)** Biolayer interferometry (BLI) trace of NCOA4^383−522^ binding to (GST-GATE16)_2_ where NCOA4^383−522^ was immobilized to the probe and was incubated with increasing concentrations (0, 78, 156, 313, and 625 nM, 1.25, 2.5, and 5 μM) of (GST-GATE16)_2_. Initialization of each incubation is noted by asterisks. Trace depicted is background-corrected for binding of (GST-GATE16)_2_ alone to the probe at each concentration.

**Figure S1.**
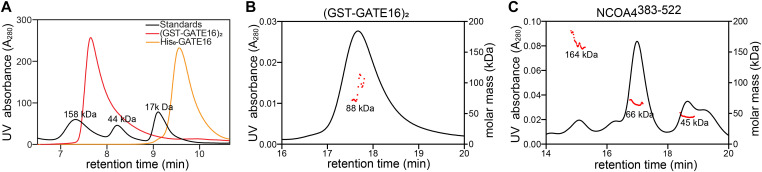
Oligomeric states of GATE16 and NCOA4^383–522^ in solution. Related to Fig 1. **(A)** Chromatographic trace monitoring absorbance at 280 nm of (GST-GATE16)_2_ and His_6_-GATE16. Molecular weight standards (black) annotated. Note that monomeric His_6_-GATE16 has an expected molecular weight of 15 kD. **(B)** SEC-MALS analysis of (GST-GATE16)_2_. Black curve traces absorbance at 280 nm. Red dots represent determined molar mass across peak. Measured mass of peak is 88 ± 14 kD. Note that dimeric GST-GATE16 has an expected molecular weight of 80 kD. **(C)** SEC-MALS analysis of NCOA4^383–522^. Black curve and red dots as in (B). Measured masses from peaks left to right are 164 ± 7.3 kD, 66.2 ± 2.0 kD, and 44.8 ± 2.0 kD, respectively. Note that monomeric AviTag-His6-NCOA4^383–522^-MBP construct used in all in vitro assays has an expected molecular weight of 61 kD and that isolated MBP has a molecular weight of 40 kD.

**Figure S2.**
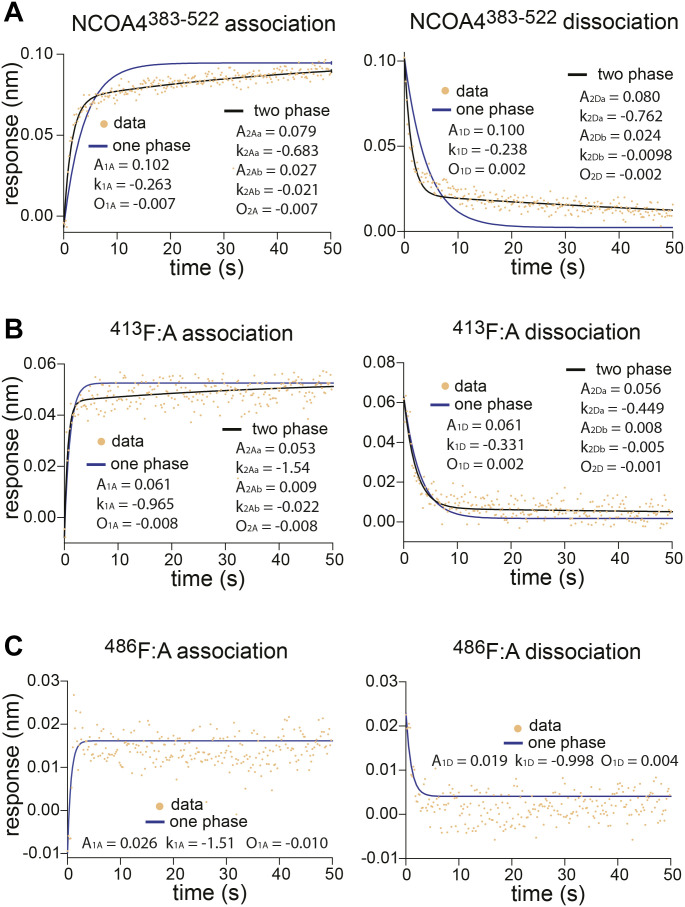
NCOA4^383–522^ and (GST-GATE16)_2_ binding kinetics measured by BLI. Related to Fig 1. **(A)** BLI association (left) and dissociation (right) curves of NCOA4^383–522^ binding to 5 μM (GST-GATE16)_2_. BLI response data fit in Prism to one- and two-phase models as follows: A_1A_ (1-e^k1A*t^) + O_1A_ | one-phase association A_1D_e^k1D*t^ + O_1D_ | one-phase dissociation. A_2Aa_(1-e^k2Aa*t^) + A_2Ab_(1-e^k2Ab*t^) + O_2A_ | two-phase association. A_2Da_e^k2Da*t^ + A_2Db_e^k2Db*t^ + O_2D_ | two-phase dissociation. **(B)** BLI response curves of ^413^F:A binding fit as in (A). **(C)** BLI response curves of ^486^F:A binding fit as in (A).

**Figure S3.**
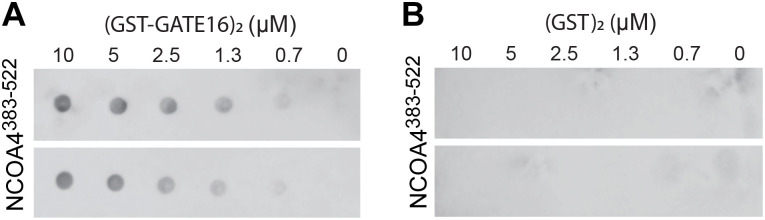
Assessment of nonspecific binding of NCOA4^383–522^ to (GST)_2_ in Far-Westerns. Related to Fig 1. **(A)** Far-Western blot of NCOA4^383–522^ binding to (GST-GATE16)_2_, which was spotted on the membrane at the indicated concentration. NCOA4^383–522^ incubated on the membrane at 0.1 μM. Signal detected by HRP-conjugated streptavidin. Two replicates are shown, with the lower replicate reproduced from Fig 1C. **(B)** Far-Western as in (A), with GST replacing (GST-GATE16)_2_.

**Figure S4.**
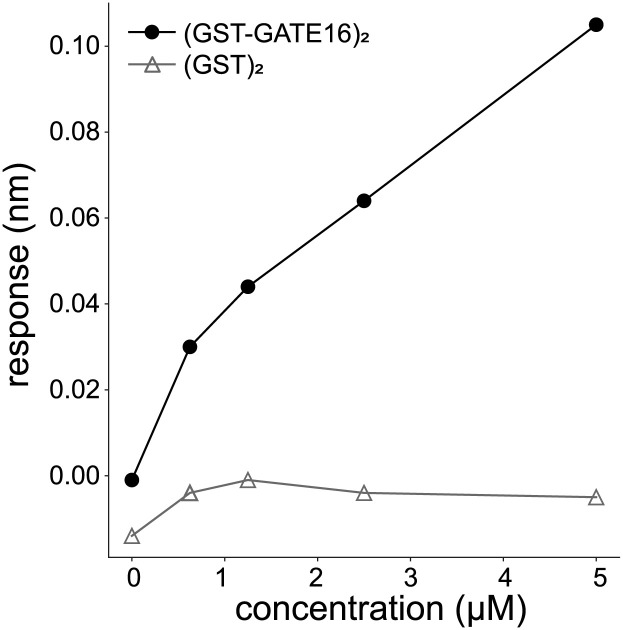
Assessing the nonspecific association with NCOA4^383–522^ to (GST)_2_. Related to Fig 1. BLI response measurement of NCOA4^383–522^ binding to either (GST-GATE16)_2_ or to isolated (GST)_2_ where the NCOA4^383–522^ construct was immobilized to the probe and tested against increasing concentrations (0 and 625 nM; 1.25, 2.5, and 5 μM) of either construct. (GST-GATE16)_2_ curve reproduced from Fig 2B.

**Figure S5.**
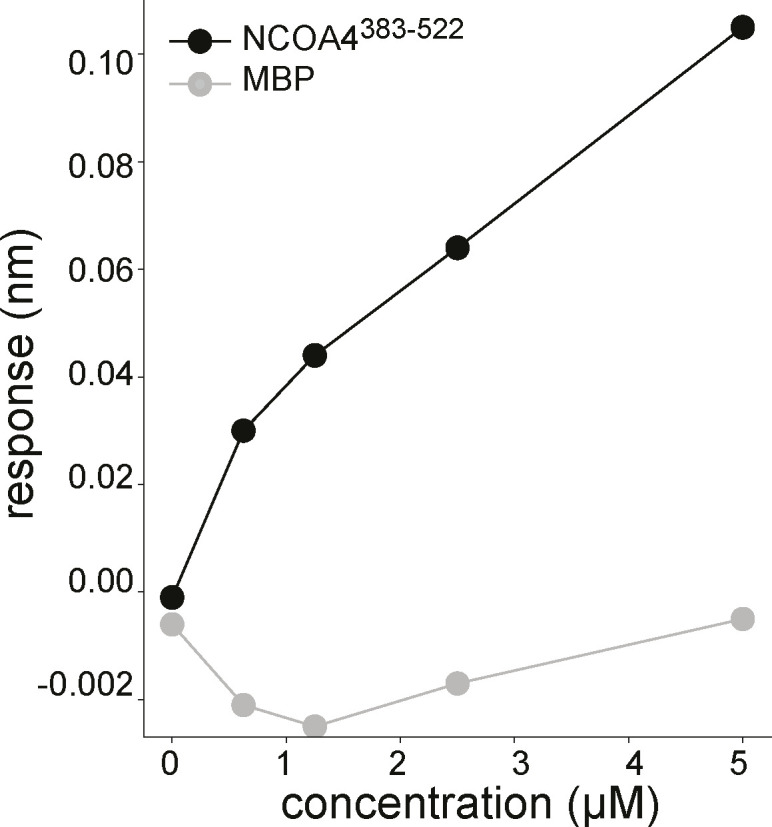
Assessing the nonspecific association of MBP to (GST-GATE16)_2_ in BLI. Related to Fig 1. BLI response measurement of AviTag-His_6_-NCOA4^383–522^-MBP (NCOA4^383–522^) or MBP binding to (GST-GATE16)_2_ where NCOA4^522^ or MBP was immobilized to the probe and tested against increasing concentrations (0 and 625 nM; 1.25, 2.5, and 5 μM) of (GST-GATE16)_2_. The response height was measured at each concentration. The NCOA4^383–522^ curve reproduced from Fig 2B.

Though it has been previously reported that NCOA4 interacts with GATE16, this interaction was exclusively demonstrated through co-immunoprecipitations from cell lysates and cellular colocalization studies with full-length NCOA4 (Dowdle et al, 2014; Mancias et al, 2014). Such evidence does not preclude indirect GATE16•NCOA4 binding through a protein intermediate. Thus, we sought to determine whether NCOA4 and GATE16 can, in fact, bind directly. For these experiments, we used the biochemically amenable fragment of NCOA4^383−522^ (Fig 1A and B) that is sufficient to bind ferritin (Hoelzgen et al, 2024), but lacks both the putative coiled-coil domain and domains that overlap with the β-isoform, which does not bind ferritin or support ferritinophagy (Mancias et al, 2015). After purifying NCOA4^383−522^ and GST-GATE16 that, in our assay, were monomeric and dimeric, respectively (Fig S1A–C), we assessed binding via Far-Western blots and biolayer interferometry (BLI). We observed concentration-dependent association in both assays (Figs 1C and D, S2, S3A and B, S4, and S5), consistent with NCOA4^383−522^ having directly bound to this dimeric (GST-GATE16)_2_. Notably, careful inspection of the BLI traces revealed biphasic binding kinetics in the association and dissociation phases, consistent with the presence of at least two binding events (Fig S2A–C).

### NCOA4^383−522^ binds to GATE16 via two LIR-like motifs

**Figure S6.**
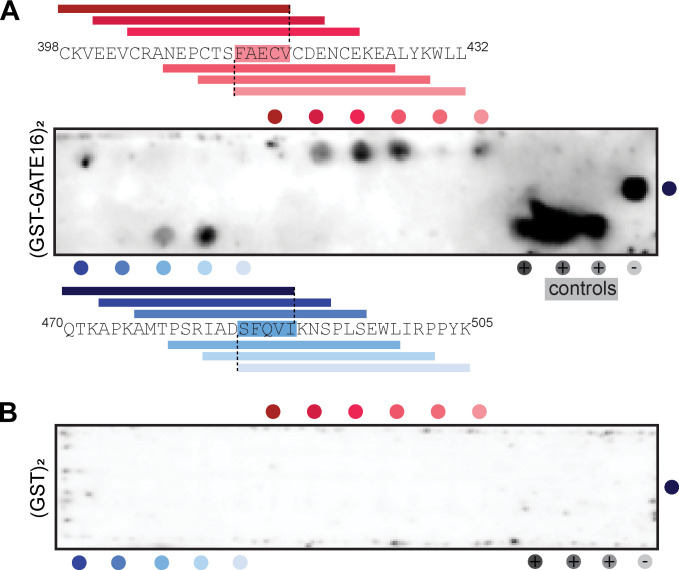
NCOA4^383–522^ binding to (GST-GATE16)_2_ via peptide array. Related to Fig 2. **(A)** Binding of (GST-GATE16)_2_ to a peptide spot array composed of 20mers tiled across the NCOA4^383–522^ sequence, where each peptide is offset three amino acids. Peptides are arrayed N-to-C in rows (3) from left-to-right. Positive and negative controls at the right in the third row. (GST-GATE16)_2_ binding was detected using an HRP-conjugated anti-GST antibody. Sites of (GST-GATE16)_2_ binding are indicated by colored dots adjacent to the peptide array, with corresponding peptide sequence spans depicted. Positive control peptides arrayed left-to-right are noted with grey circles and were derived from p62/SQSTM (SGGDDDWTHLSS), ATG4B (EDEDFEILSL), and FYCO1 (DDAVFDIITDEELCQIQE), with known LIR motif underlined. Negative control peptide noted by light gray circle corresponded to a poly-His peptide (HHHHHHGSSHHHHHHGSSHH). **(B) **Far-Western as in (A), with GST replacing (GST-GATE16)_2_.

**Figure S7.**
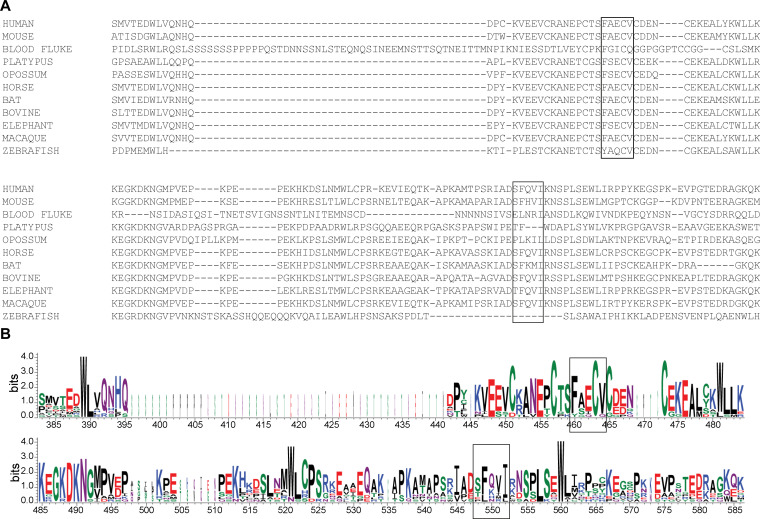
Sequence conservation of the two LIR-like motifs of NCOA4^383–522^. Related to Fig 2. **(A)** Sequence alignment of human NCOA4^383–522^ and orthologs. Identified LIR-like motifs are indicated with boxes. Alignment generated using ClustalW (EMBL-EBI) with default settings. **(B)** Sequence logo of the alignment in (A) generated by WebLogo 3.7.4 (Crooks et al, 2004). Motifs are indicated with boxes. Height at each position indicates the overall sequence conservation at that position and height of individual letters at each position indicates the relative frequency of the residue for that position. The width of each position is proportional to the number of aligned sequences at that position.

**Figure 2.**
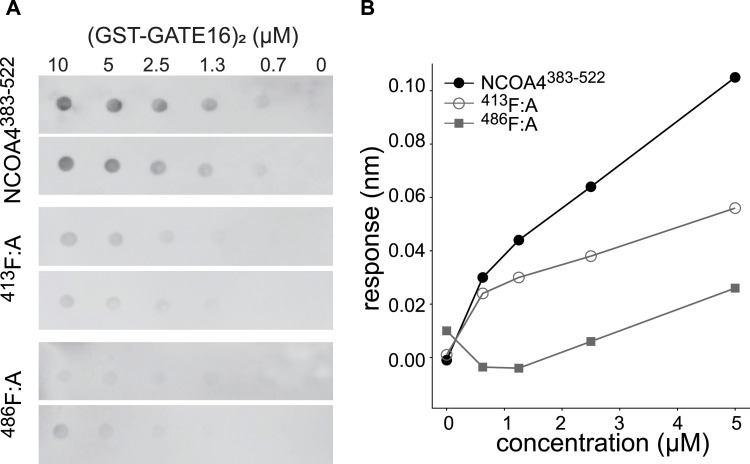
NCOA4^383−522^ binds to (GST-GATE16)_2_ through two LIR-like motifs. **(A)** Far-Western of NCOA4^383−522^ and NCOA4^383−522^ motif mutants binding to (GST-GATE16)_2_, which was spotted on the membrane at indicated concentrations. Binding was detected using HRP-conjugated streptavidin directed to Avi-tagged NCOA4^383−522^, which was incubated on the membrane at a concentration of 0.1 μM. NCOA4^383−522^ and motif mutants were assayed in duplicate. **(B)** BLI response curve of NCOA4^383−522^ and motif mutants binding to (GST-GATE16)_2_, where the NCOA4^383−522^ construct was immobilized to the probe and tested against increasing concentrations (0 and 625 nM, 1.25, 2.5, and 5 μM) of (GST-GATE16)_2_. FAECV motif mutant noted as ^413^F:A and SFQVI mutant labeled as ^486^F:A, where Phe in each motif was mutated to Ala.

**Figure S8.**
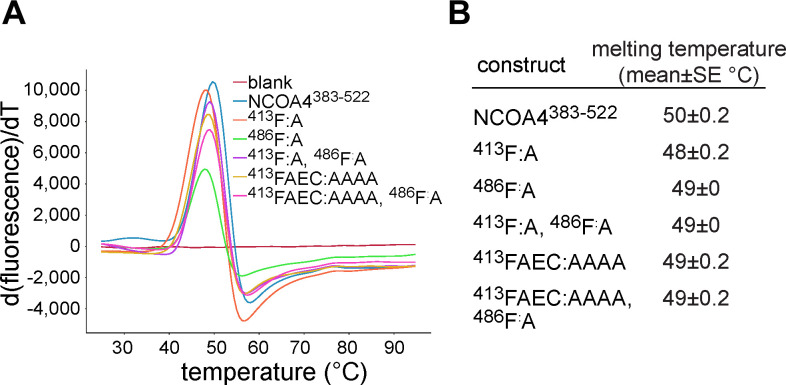
Impact of NCOA4^383–522^ mutants on protein stability. Related to Fig 2. **(A)** Protein thermal shift assay melting curves plotting the first derivative of SYPRO Orange fluorescence emission as a function of temperature for NCOA4^383–522^ and NCOA4^383–522^ mutants (see the Materials and Methods section). **(B)** Table of melting temperature for NCOA4^383–522^ and NCOA4^383–522^ mutants measured in (A). Mean and standard error of three replicates for each construct listed.

NCOA4^383−522^ contains several putative LIR-like sequence motifs. To determine whether such motifs supported GATE16•NCOA4 binding, we constructed a tiled array of peptides, each 20 amino acids long, that spanned the NCOA4^383−522^ sequence, and we probed this array for binding with (GST-GATE16)_2_. The peptide array revealed two candidate linear interacting regions within the NCOA4 fragment—specifically ^413^FAECV^417^ and ^485^SFQVI^489^ (Fig S6A and B). Of note, whereas the ^485^SFQVI^489^ motif contained a canonical LIR sequence, the ^413^FAECV^417^ motif bore cysteine substituted for the L/I/V residue specified by the canonical four-residue LIR motif. We found that these identified motifs were highly conserved among NCOA4 orthologs, with the limited observed sequence variation preserving the hydrophobic nature of the key residues, including this noted cysteine (Fig S7A and B). We note that these regions are also reported to support binding to HERC2 and ferritin, which could in addition explain the observed patterns of conservation (Hoelzgen et al, 2024; Liu et al, 2025). To directly assess the impact of these motifs on NCOA4^383−522^•GATE16 binding, we individually substituted alanine for the key aromatic residues (^413^F:A; ^486^F:A) in the identified LIR motifs in NCOA4^383−522^ and tested the impact of these mutations on (GST-GATE16)_2_ binding using both Far-Western blots and BLI (Fig 2A and B). In each assay, mutations to these putative binding motifs decreased (GST-GATE16)_2_ binding, consistent with the necessity of each motif. Notably, the effect was not due to simply unfolding NCOA4^383−522^, as the mutations did not impact global protein stability as assayed by a SYPRO Orange–based protein thermal stability shift assay (Fig S8A and B).

### NCOA4•GATE16 binding relies on avidity in vitro

**Figure S9.**
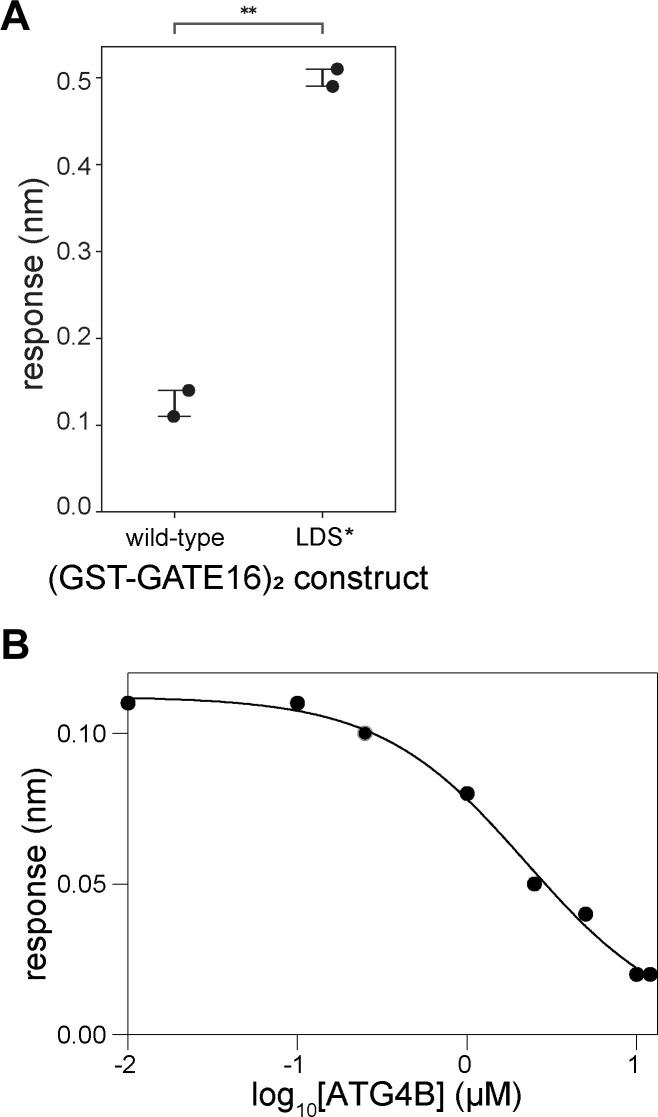
Contribution of LIR docking site (LDS) to NCOA4^383–522 ^binding to (GST-GATE16)_2_. Related to Fig 3. **(A)** BLI-based quantification of NCOA4^383–522^ binding to 5 μM WT (GST-GATE16)_2_ or LDS* (GST-GATE16)_2_, with increased response indicating increased binding. Dots denote replicate measurements, with bars marking the standard error of the mean. Statistical significance was calculated using an independent t-test (ns: *P* < 1, *P < 0.05, **P < 0.01, ***P < 0.001, ****P < 0.0001). **(B)** BLI response curve of NCOA4^383–522^ binding to (GST-GATE16)_2_ where NCOA4^383–522^ construct is immobilized to the probe and tested against 5 μM (GST-GATE16)_2_ in the presence of increasing concentrations of ATG4B peptide (EDEDFEILSL) (10, 100, and 250 nM; 1, 2.5, 5, and 10 μM). Data fit to: Y = (max − min)/(1 + 10^([ATG4B] − log10[IC50])^) + min with fit parameters: max = 0.11, min = 0.02, IC50 = 2.2 μM.

**Figure 3.**
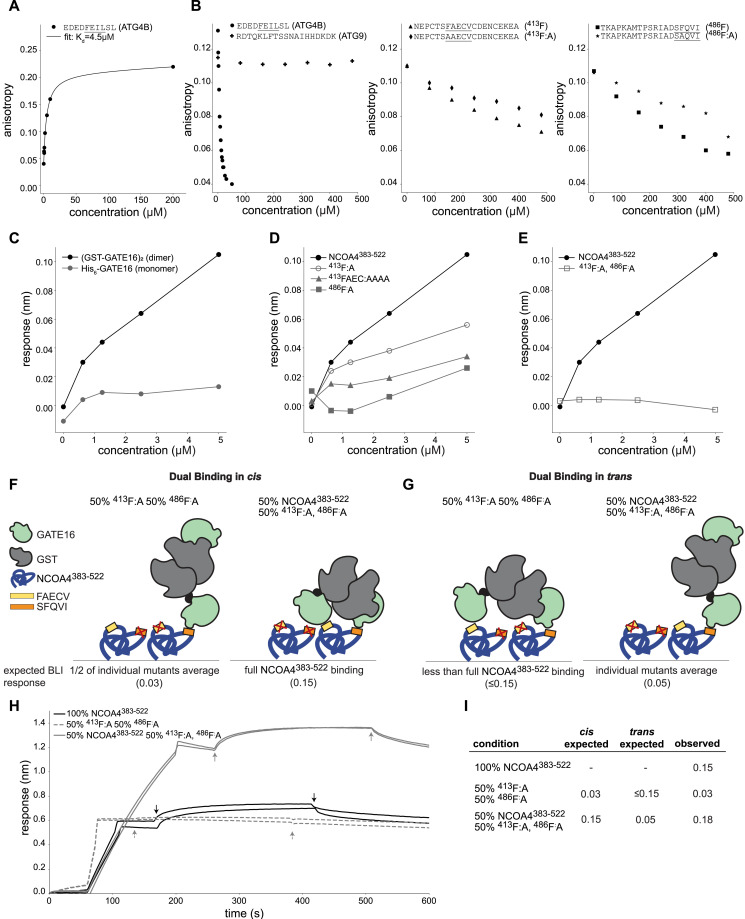
Binding of NCOA4^383−522^ to (GST-GATE16)_2_ requires avidity. **(A)** Fluorescence anisotropy of dye-labeled peptide containing the ATG4B LIR peptide (EDEDFEILSL) binding to His_6_-GATE16. Data fit to standard binding isotherm with apparent K_D_ noted. **(B)** Competition fluorescence anisotropy assay of dye-labeled ATG4B LIR peptide (EDEDFEILSL) competing for binding to monomeric His_6_-GATE16 against increasing concentrations of unlabeled peptides derived from ATG4B, ATG9, NCOA4^383−522^, or NCOA4^383−522^ motif mutants, as noted. **(C)** BLI response curve of NCOA4^383−522^ binding to His_6_-GATE16 or (GST-GATE16)_2_ where the NCOA4^383−522^ construct is immobilized to the probe and tested against increasing concentrations (0 and 625 nM, 1.25, 2.5, and 5 μM) of either GATE16 construct. **(D, E)** BLI assay of (GST-GATE16)_2_ binding to noted NCOA4^383−522^ constructs, probed at (GST-GATE16)_2_ concentrations as described in (C). **(F, G)** Schematic of hypothesized binding modes for (GST-GATE16)_2_ binding in “cis” (F) or in “trans” (G) to NCOA4^383−522^. BLI response expected for each model at the noted ratios of each protein construct is detailed in the below schematic. **(H)** BLI traces of assays performed as depicted in (F, G). Two replicates of each assay are shown. Each assay used 5 μM (GST-GATE16)_2_. **(I)** Expected responses for cis and trans models and quantification of response for BLI traces in (G).

Having identified two motifs in NCOA4^383−522^ that were each necessary for GATE16 association, we next sought to determine where on GATE16 those peptides bound. Because LIR motifs typically bind to LC3/GABARAP-family proteins in regions known as LIR docking sites (LDS) (Wirth et al, 2019; Johansen & Lamark, 2020), we hypothesized the LDS was a likely binding location. To assess this, we first mutated known key LDS residues Y49 and L50 of (GST-GATE16)_2_ to alanines (LDS*, hereafter) and tested binding to NCOA4^383−522^ via BLI. Interestingly, these mutations improved binding of GATE16 to NCOA4^383−522^ (Fig S9A). To further test whether NCOA4^383−522^ interacts with the LDS, we probed the ability of a peptide derived from ATG4B that is known to bind to the LDS (Skytte Rasmussen et al, 2017) to disrupt the binding of NCOA4^383−522^ to (GST-GATE16)_2_ in BLI. The ATG4B peptide inhibited NCOA4^383−522^•GATE16 binding in a concentration-dependent manner, further indicating that NCOA4^383−522^ is interacting with the LDS of GATE16 (Fig S9B). In addition, we performed competitive fluorescence anisotropy assays in which an unlabeled peptide containing either of the identified NCOA4^383−522^ motifs was added to a solution bearing monomeric GATE16 and a fluorescently labeled ATG4B peptide. Though the NCOA4^383−522^-derived peptides displaced the ATG4B peptide in a sequence-dependent manner, consistent with competitive binding to the LDS, they did so with weak apparent affinity (Fig 3A and B).

The observed discrepancy in NCOA4 affinity for monomeric GATE16 and dimeric (GST-GATE16)_2_ led us to hypothesize that the two LIR motifs avidly facilitated NCOA4•GATE16 binding. In our model, we reasoned that binding of one LIR-like motif to a dimerized copy of GATE16 could increase the effective concentration of the second LIR-like motif, thereby facilitating its binding to the GST-dimerized GATE16 copy. To more directly test this model, we assayed the binding of NCOA4^383−522^ to GATE16 as a function of GATE16’s oligomeric state using either monomeric His_6_-GATE16 or dimeric (GST-GATE16)_2_ (Fig S1A–C). Our BLI-based binding assay showed improved binding with (GST-GATE16)_2_ relative to monomeric His_6_-GATE16 (Fig 3C). Of note, GST alone was unable to bind NCOA4^383−522^ in any of our BLI, Far-Western, or peptide array assays (Figs S3, S4, and S6).

To further assess the impact of multiple NCOA4^383−522^ motifs on GATE16 binding, we generated single and double mutants of the identified motifs. Mutation of either motif alone significantly decreased binding to (GST-GATE16)_2_ as assayed by BLI, though neither was sufficient to completely abrogate binding (Fig 3D). Notably, replacing the FAEC of the FAECV motif with a tetra-alanine linker further abrogated binding relative to the ^413^F:A mutant, likely because of binding contribution of the cysteine residue (Fig 3D). In the double mutant (^413^F:A, ^486^F:A), we observed even weaker binding, consistent with each motif contributing to the overall NCOA4^383−522^•GATE16 interaction (Fig 3E).

**Figure S10.**
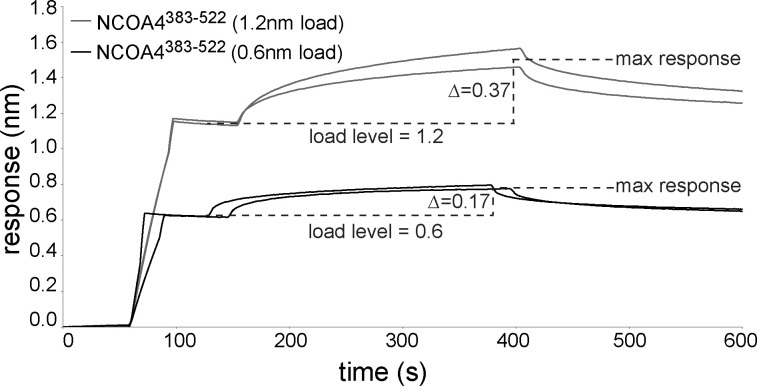
Load-dependent BLI response of (GST-GATE16)_2_ binding to NCOA4^383^–^522^. Related to Fig 3. BLI association and dissociation curves of NCOA4^383–522^ binding to 5 μM (GST-GATE16)_2_, where the NCOA4^383–522^ construct was immobilized to the probe. Replicates at NCOA4^383-522^ loading levels of 0.6 and 1.2 nm are shown. Average BLI response upon (GST-GATE16)_2_ binding measured across replicates denoted as difference (Δ) of max response and load level.

We next assessed the oligomeric state of NCOA4^383−522^ in the context of our observed NCOA4^383−522^•GATE16 binding to obtain a more complete binding model. In contrast to previous studies reporting NCOA4^383−522^ can dimerize despite lacking the N-terminal coiled-coil oligomerization domain contained in the full-length protein (Gryzik et al, 2017), we found that NCOA4^383−522^ was monomeric at our working concentrations, as assessed by SEC-MALS (Fig S1C). However, because the BLI binding assays require that NCOA4^383−522^ be immobilized on an assay probe tip, it was still formally possible that NCOA4^383−522^ was positioned in such a way that it was acting as an artificial dimer. To assess whether NCOA4^383−522^ was acting as a monomer or a dimer in this assay, we performed two complementary tests via BLI, probing for NCOA4^383−522^ binding to (GST-GATE16)_2_ either in “cis” to monomeric NCOA4^383−522^, or in “trans” to an apparently multimeric form (Fig 3F and G). In the first assay, the two individual motif mutants (^413^F:A and ^486^F:A) were mixed in a 1:1 ratio, loaded on the probe, and their binding to (GST-GATE16)_2_ was measured. If NCOA4^383−522^ were acting as a dimer in this assay, we expected binding similar to NCOA4^383−522^ lacking motif mutations, as each singly mutated NCOA4^383−522^ would still have one motif available to bind to one subunit of the GST-GATE16 dimer. Instead, we observed strongly reduced binding (Fig 3H and I). In a complementary assay, we mixed NCOA4^383−522^ and the double mutant (^413^F:A, ^486^F:A) in a 1:1 ratio, loaded the probe at twofold higher density, and measured binding to (GST-GATE16)_2_. Of note, loading the probe with twice the amount of NCOA4^383−522^ produced a proportional increase in observed binding response (Fig S10). As such, if NCOA4^383−522^ were acting as a monomer in this assay, then we would expect this protein mixture loaded at the twofold higher density to produce similar binding to that observed with the mutation-free type protein loaded at the standard density. We observed binding at a level predicted by the monomeric model (Fig 3F) and thus concluded that a monomeric NCOA4^383−522^ uses two binding motifs to associate with dimeric GST-GATE16 (Fig 3H and I).

### NCOA4^383−522^ requires two LIR-like motifs to direct lysosomal degradation of ferritin in vivo

**Figure S11.**
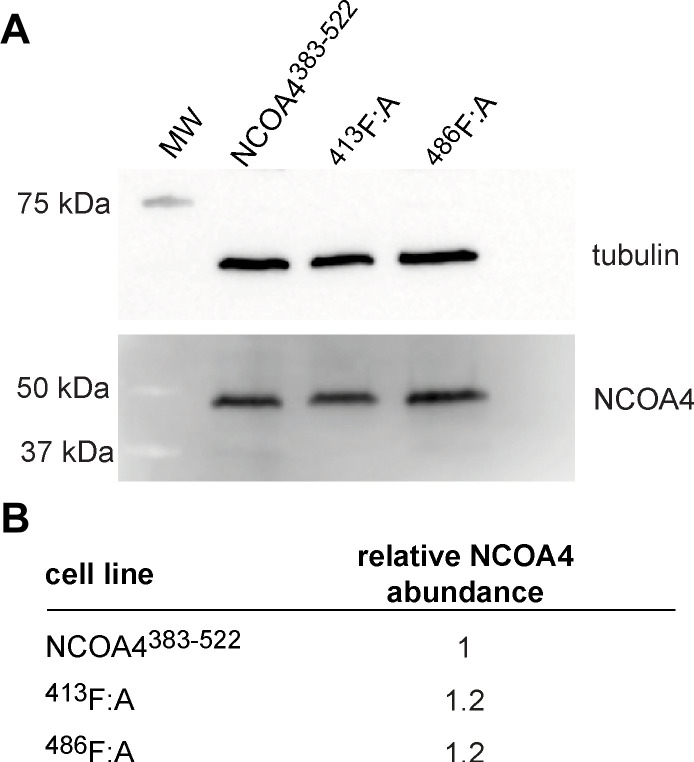
Cellular levels of NCOA4^383-522^ and associated mutants. Related to Fig 4. **(A)** Western blot assessing levels of NCOA4^383–522^, ^413^F:A, and ^486^F:A expressed under steady-state conditions in HeLa cell lines. NCOA4^383–522^ levels probed using an anti-GFP antibody against the GFP-fused NCOA4^383–522^. Tubulin levels were also probed by Western blot to assess relative sample loading. Molecular weight markers are annotated. **(B)** Table reporting NCOA4 abundance of each mutant relative to NCOA4^383–522^ as measured in (A). In each instance, levels were normalized using the tubulin loading control.

**Figure 4.**
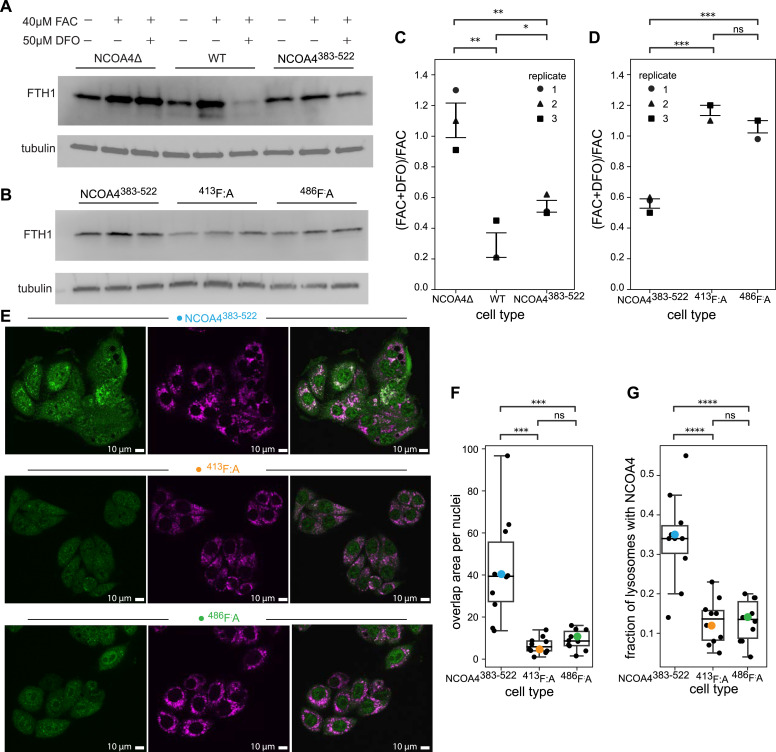
NCOA4^383−522^ supports ferritin degradation in vivo and requires both LIR-like motifs. **(A)** Western blots against ferritin heavy chain (FTH1) and tubulin in NCOA4Δ, WT, and NCOA4^383−522^ cell lines. Cells were either untreated, treated for 24 h with ferric ammonium citrate (FAC), or treated with FAC before undergoing a 24-h treatment with deferoxamine (DFO). **(B)** Western blots as described in (A) in either NCOA4^383−522^, ^413^F:A, or ^486^F:A cell lines. **(C, D)** Quantification of triplicate ferritin degradation assays described in (A, B). Bars denote the standard error of the mean. The fraction of ferritin degraded was calculated in FIJI (Schindelin et al, 2012) using condition-dependent band intensities as follows: ([FAC + DFO]_FTH1_/[FAC + DFO]_tubulin_)/(FAC_FTH1_/FAC_tubulin_). Statistical significance was calculated via an independent *t* test (ns: *P* < 1, *P < 0.05, **P < 0.01, ***P < 0.001). **(E)** Representative images probing for colocalization of GFP-NCOA4^383−522^ and lysosomes, as detected with an anti-LAMP1 antibody, in NCOA4^383−522^ (top panels), ^413^F:A (middle panels), or ^486^F:A (bottom panels) cell lines treated with 50 μM DFO. The anti-GFP signal shown in green is in panels at left, anti-LAMP1 signal shown in pink is in middle panels, and a merged image with overlapping areas shown in white is in the panels at right. 10 μm scale bar shown in white. **(F)** Boxplot of colocalization assay described in (E), with colocalization quantified as a function of area of overlap per nucleus. Black dots represent individual images, with colored dots corresponding to images in (E). Significance was calculated via an independent *t* test (ns: *P* < 1, *P < 0.05, **P < 0.01, ***P < 0.001, ****P < 0.0001). **(G)** Boxplot of colocalization assay described in (E), with colocalization quantified as a function of fraction of lysosomes containing NCOA4^383−522^. Values were calculated as Manders’ coefficients in FIJI. Black dots represent individual images, with colored dots corresponding to images in (E). Significance was calculated via an independent *t* test (ns: *P* < 1, *P < 0.05, **P < 0.01, ***P < 0.001, ****P < 0.0001).

Having established the importance of these two LIR-like motifs in vitro, we next assessed the role of these motifs in vivo. To do so, we stably integrated NCOA4^383−522^ or variants mutated in the LIR-like motifs into NCOA4 knockout HeLa cells (NCOA4Δ, hereafter) (Gryzik et al, 2021). We measured similar expression levels of NCOA4 in each transfected cell line (Fig S11A and B). With these cell lines, we first determined whether the NCOA4^383−522^ fragment could facilitate the degradation of ferritin by comparing the extent of ferritin degradation in WT, NCOA4Δ, and NCOA4^383−522^ cells upon iron chelation with deferoxamine (DFO). Using quantitative Western blots (see the Materials and Methods section), we observed that ferritin was not degraded in NCOA4Δ cells, whereas ferritin degradation was robust in WT cells and those bearing the NCOA4^383−522^ (Fig 4A and C). Notably, the WT cells exhibited greater chelation-induced degradation than those expressing the NCOA4^383−522^ fragment, suggesting additional contributions to ferritinophagy outside of this biochemically amenable NCOA4 fragment.

Given that NCOA4^383−522^ facilitated ferritin degradation, we next assessed the impact of mutations to the two LIR-like motifs. Consistent with our in vitro binding data, mutation of either motif was sufficient to prevent NCOA4^383−522^-dependent ferritin degradation (Fig 4B and D), supporting the hypothesis that both motifs are required for stable NCOA4^383−522^ binding to LC3/GABARAP-family proteins and by extension for ferritin degradation in cells.

Finally, to assess whether the observed ferritin degradation occurred in the lysosome, we performed colocalization studies via immunofluorescence to measure the impact of these identified LIR motifs on NCOA4:lysosome colocalization. Specifically, we stained for GFP-NCOA4^383−522^ and lysosomes (LAMP1) in our NCOA4^383−522^ and NCOA4^383−522^ mutant cell lines in the presence of DFO. We observed significant colocalization of NCOA4^383−522^ with lysosomes, consistent with NCOA4-mediated ferritin degradation through the autophagy–lysosomal pathway (Fig 4E). Furthermore, we found that mutation of either NCOA4^383−522^ LIR-like motif resulted in significantly decreased NCOA4:lysosome colocalization (Fig 4F and G).

**Figure S12.**
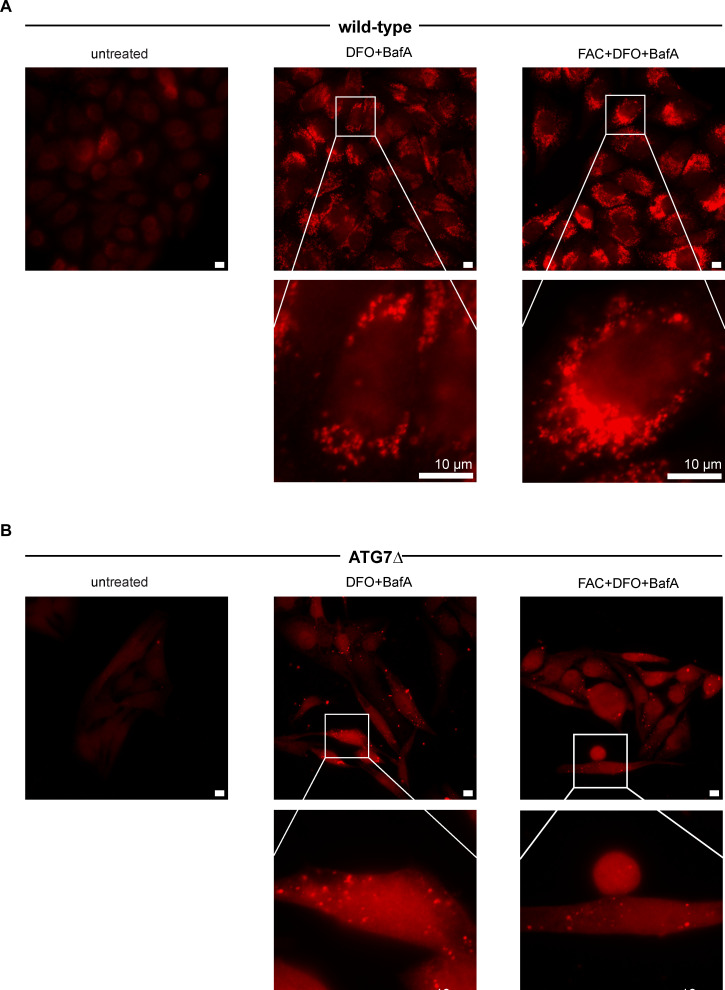
LC3B form puncta independent of ATG7 in ferritinophagy-inducing conditions. Related to Fig 4. **(A)** Representative images for wild-type HeLa cells untreated, treated with 40 μM FAC, 50 μM DFO, or 400 nM BafA, as indicated (see the Materials and Methods section). Anti-LC3B signal is shown in red. 10 μm scale bar in white in full image and enlarged inset. **(B)** Images as described in (A) for ATG7Δ HeLa cells.

The dependence of NCOA4:lysosome colocalization on both NCOA4^383−522^ LIR-like motifs further indicated a role of avidity in the observed activity, and by extension binding of NCOA4^383−522^ to oligomerized GATE16, in vivo. However, some previous studies have shown that ferritinophagy can occur in cells lacking the LC3 lipidation machinery (ATG7), which would preclude membrane-facilitated multimerization of LC3 family proteins (Goodwin et al, 2017; Kuno et al, 2022). Hence, we sought to test whether LC3s oligomerize independently of ATG7. To do so, we probed the ability of both WT and ATG7 knockout (ATG7Δ, hereafter) HeLa cells to form LC3B puncta. Both cell lines formed puncta in DFO and FAC + DFO conditions, albeit ATG7Δ to a much lesser degree, suggesting that LC3s can concentrate locally independent of ATG7-dependent lipidation under ferritinophagy-inducing conditions (Fig S12A and B).

### Iron regulates the binding of NCOA4^383−522^ to GATE16

**Figure 5.**
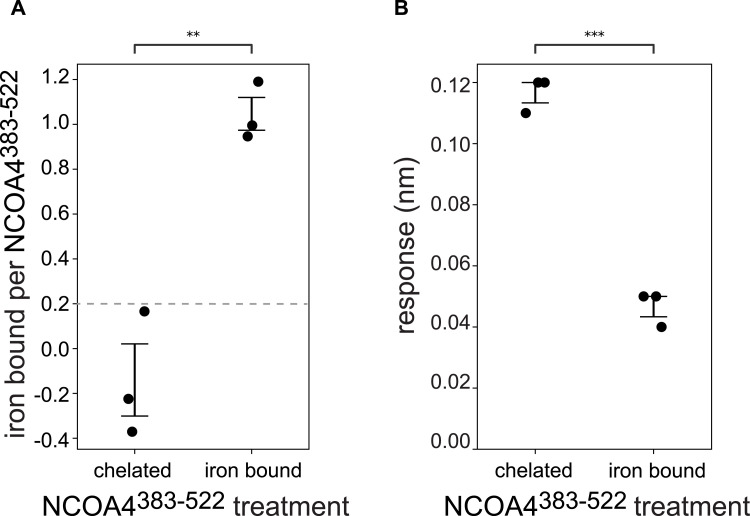
NCOA4^383−522^•(GST-GATE16)_2_ binding is iron-dependent. **(A)** Quantification of iron bound per monomer of NCOA4^383−522^ for chelated and reconstituted (i.e., iron-bound) NCOA4^383−522^ as measured in a ferene assay (Fish, 1988; McCarthy & Booker, 2018; Levitz et al, 2022). Each sample was assayed in triplicate, and the lower limit of quantitation is noted with a dashed line. Statistical significance was calculated via an independent *t* test (ns: *P* < 1, *P < 0.05, **P < 0.01, ***P < 0.001). **(B)** Quantification of chelated and iron-bound NCOA4^383−522^ binding to 10 μM (GST-GATE16)_2_ represented as response change in BLI. Each sample was assayed in triplicate. Statistical significance was calculated via an independent *t* test as in (A), with asterisks noting statistical significance as described.

Given that the NCOA4•GATE16 interaction is crucial in the progression of ferritinophagy, an iron-sensing pathway, we next asked whether iron could regulate this interaction. It was recently reported that NCOA4^383−522^ can bind an iron-sulfur cluster coordinated by four cysteines (Zhao et al, 2024; Liu et al, 2025). Importantly, one of these four cysteines, residue 416, is present in the ^413^FAECV^417^ LIR-like motif described here. Thus, we hypothesized that were NCOA4^383−522^ iron-loaded, the FAECV motif would be occluded, which would prevent robust GATE16 binding. To test this, we chelated iron from purified NCOA4^383−522^, or reconstituted the protein with iron anaerobically (see the Materials and Methods section). We used a ferene assay (Fish, 1988; McCarthy & Booker, 2018; Levitz et al, 2022) to measure the equivalents of iron bound in each sample (Fig 5A). For each sample, we then measured binding to (GST-GATE16)_2_, observing a marked decrease in (GST-GATE16)_2_ binding in the iron-bound NCOA4^383−522^ sample (Fig 5B), consistent with a role of iron in negatively regulating NCOA4^383−522^•GATE16 binding.

## Discussion

Taken together, our biochemical study revealed that NCOA4^383−522^, a biochemically amenable fragment of the selective autophagy receptor for ferritin, can bind directly to GATE16 through two LIR-like motifs: ^413^FAECV^417^ and ^485^SFQVI^489^. We found that in isolation, each motif binds GATE16 weakly and that NCOA4^383−522^ relies on avidity between these motifs to achieve tight binding. Moreover, we observed that to robustly form this complex, GATE16 must be oligomeric, though NCOA4^383−522^ need not be. Our work further showed that the NCOA4^383−522^ fragment is sufficient for ferritinophagy and that each LIR-like motif is indispensable for this activity in vivo. Finally, we found that binding of iron to NCOA4^383−522^ reduces its affinity for GATE16, providing a mechanistic link between cellular iron levels and targeted ferritinophagy.

Work over the prior 15 yr has resulted in an ever-expanding collection of canonical and noncanonical peptide sequences that support binding to LC3/GABARAP-family proteins (Johansen & Lamark, 2020; Chatzichristofi et al, 2023). One of the peptides we identified (^485^SFQVI^489^) follows the canonical LIR pattern (i.e., [W/F/Y]-x-x-[I/L/V]), whereas the other (^413^FAECV^417^) is, to our knowledge, the first instance of a cysteine acting as a core hydrophobic residue in a mammalian LIR. By some measures, reduced cysteine, when protonated, is hydrophobic and often found in the core of proteins (Nagano et al, 1999), making it a reasonable candidate to occupy one of the hydrophobic pockets on GATE16. Combining this and other observed noncanonical residues at this position (Kosmatka et al, 2026 *Preprint*; Farnung et al, 2023) with structures demonstrating flexibility in the number of residues separating the two core LIR residues (Knaevelsrud et al, 2013; Keown et al, 2018) leads to a conclusion that the canonical LIR motif is overly stringent, and that the proteome displays an even larger array of potential LC3/GABARAP-family–interacting peptides than currently appreciated. Such an expanded understanding of this interface further highlights the challenges in achieving specificity, emphasizing the importance of our described avidity model.

In cells, LC3/GABARAP-family proteins are relatively abundant (Beck et al, 2011; Huang et al, 2023) and the canonical LIR motif of [W/F/Y]-X-X-[I/L/V] is of low information content and thus common in the human proteome. How then do cells achieve the high binding selectivity one would expect given the degradative capacity of the autophagy–lysosomal pathway? Whereas additional selectivity determinants outside of the core LIR, including a previously described acidic patch N-terminal of the core LIR (Birgisdottir et al, 2013; Johansen & Lamark, 2020), likely play a role, our work in addition suggests that avidly linking multiple LIR-like motifs in a single complex can contribute to specificity and affinity. Under this model, selective receptors, or complexes thereof, would need to expose multiple LIRs to support binding to LC3s, with such a multivalency requirement adding an additional layer of binding selectivity. Notably, in this model, cells could regulate whether such LIRs were displayed in a condition-specific manner, providing a direct means to modulate selective autophagy.

In support of this avidity model, we have identified two LIR-like motifs in NCOA4^383−522^ that cooperatively facilitate binding to GATE16. In this model, binding of one motif to a single subunit of (GST-GATE16)_2_ increases the local concentration of the dimer-linked GATE16 protomer, which facilitates the second binding event. As we have shown that each motif is indispensable for ferritinophagy in vivo, we hypothesize that LC3/GABARAP-family proteins must also act as oligomers to support NCOA4 binding in cells. Although our work has focused on GATE16 binding specifically, NCOA4 is thought to interact with multiple members of the LC3/GABARAP family (Mancias et al, 2014), which would allow for avid interactions across the LC3/GABARAP-family members. The fact that the LC3/GABARAPs are conjugated to the autophagosomal membrane, which can situate them at high concentration on a 2D surface, offers one potential mechanism by which they could mimic an oligomeric state. However, there are reports of NCOA4-mediated ferritinophagy that does not require ATG7 lipidated LC3s in some conditions (Goodwin et al, 2017; Kuno et al, 2022). Here, we found that LC3s can form puncta in ATG7Δ cells. Consistent with this observation, Runwal et al, observed LC3 puncta in cells depleted of ATG7 and ATG10, as well as in cells bearing nonconjugatable mutants of LC3B, GABARAP, and GEC-1 in ATG16 knockout cells (Runwal et al, 2019). Taken together, this suggests that LC3/GABARAPs can concentrate locally in alternate modes that could still support avid interactions for ferritinophagy. In addition, prior reports have also cited that in some conditions, TAX1BP1, which also binds to LC3-family proteins (Zhang et al, 2024), facilitates ferritinophagy (Goodwin et al, 2017; Ohshima et al, 2022), further expanding the potential set of ferritinophagy pathways.

It has become increasingly clear that avidity plays a large role in the progression of autophagy (Zaffagnini & Martens, 2016). In addition to our proposed NCOA4•GATE16 binding model, a similar mechanism has been suggested in yeast where avidity is used to support the selective receptor Atg19 binding to Atg8, the yeast homolog of the LC3/GABARAPs (Sawa-Makarska et al, 2014), and in mammalian cells where the selective autophagy receptors p62 and OPTN were each shown to self-oligomerize to facilitate the degradation of their respective cargo (Ying et al, 2010; Wurzer et al, 2015). Moreover, at the most extreme end of low-affinity, high-avidity interactions, phase separation has been proposed to have a role in autophagic initiation and maturation (Zhang et al, 2018; Fujioka et al, 2020). Our work highlighting the role of avidity in facilitating NCOA4 binding to oligomerized LC3/GABARAPs complements these prior observations.

As the process of ferritinophagy releases iron predominantly in response to iron depletion, there are several proposed mechanisms linking ferritinophagy to levels of labile iron. In one model, in iron-replete conditions, the E3 ubiquitin ligase HERC2 binds to NCOA4 and promotes its degradation via the proteasome (Mancias et al, 2015; Zhao et al, 2024), thereby negatively regulating ferritinophagy. In addition, it has been recently shown that NCOA4 binds an iron-sulfur cluster, which is coordinated using cysteine residues 404, 410, 416, and 422, and that ablation of the iron-sulfur cluster increases NCOA4’s affinity for ferritin (Zhao et al, 2024; Liu et al, 2025). Interestingly, cysteine 416 is contained in our described GATE16-binding ^413^FAECV^417^ motif, raising the possibility of iron-sulfur–dependent regulation of this binding activity. Here, we showed that in an iron-bound state, NCOA4^383−522^’s affinity for GATE16 is greatly reduced. Hence, the presence of iron dually inhibits GATE16 and ferritin binding, and thus ferritinophagy. We postulate that when levels of labile iron fall, this iron-sulfur cluster could be removed from NCOA4, liberating the ^413^FAECV^417^ motif to bind GATE16 and facilitate ferritinophagy, effectively adding an additional layer of iron regulation to the ferritinophagy pathway.

## Materials and Methods

### Protein expression and purification

GST-tagged GATE16 was expressed from the plasmid pGex-4T-2_GATE-16 (73518; pl_JD338; Addgene) in *Escherichia coli* BL21 Tuner DE3 (st_JD494). His_6_-tagged GATE16 was generated via Q5 site-directed mutagenesis PCR (New England Biolabs), replacing the GST tag with the sequence MHHHHHHGS, and this construct (pl_JD339) was expressed in *E. coli* strain st_JD494. The LDS* mutant of GST-GATE16 was generated by mutating Y49 and L50 to alanines using Q5 site-directed mutagenesis (NEB). A g-block gene fragment (IDT) containing the sequence for NCOA4^383−522^ was cloned into the plasmid pDW363 (8842; Addgene) via HiFi assembly (New England Biolabs) such that it had an N-terminal AviTag followed by a His_6_ tag and a C-terminal MBP (pl_JD337). This vector co-expresses the biotin ligase BirA, enabling NCOA4^383−522^ biotinylation of its AviTag during expression. Mutants of NCOA4^383−522^ (^413^F:A, ^486^F:A, ^413^F:A/^486^F:A, ^413^FAEC:AAAA/^486^F:A, ^413^FAEC:AAAA; corresponding to plasmids pl_JD344–pl_JD348, respectively) were generated using Q5 site-directed mutagenesis PCR. All NCOA4^383−522^ constructs were expressed in *E. coli* strain st_JD494.

For each protein purified, expression cultures (2 liters) were grown in 2xYT media at 37°C with aeration, and induced with 1 mM isopropyl β-d-1-thiogalactopyranoside at an optical density of ∼0.6. Media for expression of NCOA4^383−522^ constructs were supplemented with 0.05 mM biotin. Expression proceeded for 3 h at 37°C for GATE16 constructs, or at 30°C for NCOA4^383−522^ constructs before cells were harvested via centrifugation.

Cell pellets bearing GST-GATE16 were resuspended in 50 ml buffer PBS (140 mM NaCl, 2.7 mM KCl, 10 mM Na_2_HPO_4_, 1.8 mM KH_2_PO_4_, pH 7.3), dounced until homogeneous, and sonicated using Qsonica Sonicator (5 s on, 10 s off for 5 min, amplitude 35%). The lysate was centrifuged at 251,000*g* for 1 h in a Ti60 rotor, after which the soluble fraction was loaded on a 20 ml glutathione-Sepharose affinity column (GSTPrep FF 16/10, Cytiva), washed with three column volumes of resuspension buffer, and eluted with five column volumes of buffer EB (50 mM Tris–HCl, 150 mM NaCl, 10 mM reduced glutathione, pH 8.0). To generate monomeric GATE16 (mGATE16) for use in fluorescence anisotropy assays, the GST tag was then removed by the addition of 20 μl thrombin (69671-3; Millipore), and the sample was cleaved at 4°C overnight. The eluate or cleaved fractions for (GST-GATE16)_2_ or mGATE16, respectively, were pooled, concentrated to 2 ml, and purified over a S75 16/600 size-exclusion column in buffer SB (20 mM Tris, 150 mM NaCl, pH 7.5). Fractions bearing (GST-GATE16)_2_ (∼0.4 CVs) or mGATE16 (∼0.7 CVs) were identified by SDS–PAGE, pooled, and concentrated via centrifugation in a 30-kD (GST-GATE16) or 3-kD (mGATE16) concentrator (UFC903024 and UFC900324; MilliporeSigma) to a stock concentration of ∼500 μM. GST was expressed and purified as described for (GST-GATE16)_2_, using expression plasmid pl_JD340.

Cell pellets bearing His_6_-GATE16 were resuspended in 50 ml of buffer NLB (10 mM K_2_HPO_4_, 300 mM NaCl, 20 mM KCl, 10 mM imidazole, 5 mM 2-mercaptoethanol, pH 8.0), lysed and clarified as above and then loaded onto a 5-ml Ni-NTA column (Bio-Rad), washed with two column volumes of buffer NLB, and eluted over 20 column volumes in a linear gradient of buffer NLB with imidazole increasing from 10 mM to 1 M. Pooled fractions totaling 18 ml were then concentrated to 2 ml using 3-kD Centricon (UFC900324; MilliporeSigma) and purified over a S75 16/600 size-exclusion column in buffer SB. Fractions bearing His_6_-GATE16 (∼0.7 CVs) were identified and concentrated as above.

Cell pellets bearing NCOA4^383−522^ were resuspended in 50 ml of buffer NLB, lysed, clarified, and purified on a Ni-NTA column as above. The eluate (∼20 ml) was then diluted fivefold in buffer SB, loaded onto a 5-ml MBPTrap HP column (Cytiva), washed with five column volumes of buffer SB, and eluted with eight column volumes of buffer SB supplemented with 10 mM maltose. Eluate was pooled, concentrated to 2 ml, and then purified over a S75 16/600 size-exclusion column in buffer SB. Fractions (∼0.4 CVs) were identified and concentrated with 30-kD Centricon (UFC903024; MilliporeSigma) to ∼50 μM.

### Far-Western blots

(GST-GATE16)_2_ at the noted concentrations was spotted (2 μl) on a nitrocellulose membrane (Amersham Protran) and allowed to dry at RT for 12 min. The membrane was then blocked in buffer TBST (20 mM Tris, 150 mM NaCl, 0.1% Tween-20, pH 7.5) supplemented with 3% BSA for 15 min at RT before being incubated with a 0.1 μM solution of the appropriate biotinylated NCOA4^383−522^ protein construct. After washing three times for 3 min with TBST, the membrane was incubated with HRP-conjugated streptavidin (21130; Thermo Fisher Scientific) diluted 1:100,000 and subsequently washed three times in TSBT as above, before applying high-sensitivity enhanced chemiluminescent substrate (34094; Thermo Fisher Scientific). All membranes being compared were imaged concurrently over a 2-min exposure on an Azure Biosystems imager.

### Biolayer interferometry (BLI)

All BLI experiments were conducted on an Octet RED96 instrument (Forte Bio) using streptavidin biosensors (18-5019; Sartorius). Streptavidin biosensors were preincubated with BLI buffer (20 mM Tris, 150 mM NaCl, 0.05% Tween-20, 1% BSA, 1 mM DTT, pH 7.5) for 10 min. Biotinylated NCOA4^383−522^ constructs were diluted to 50 nM in BLI buffer and loaded on the streptavidin biosensors to a response level of 0.6 nm. The loaded biosensors were immersed in serial dilutions of the appropriate GATE16 construct at the noted concentrations at an orbital shake speed of 1,000 rpm at 30°C for a 250-s association step, with a dissociation step in BLI buffer for 500 s between each concentration. For each experiment, background binding of GATE16 to the biosensor was measured using a biosensor lacking NCOA4, and the same process was repeated for each concentration. This background binding was subtracted from the resulting assay curves. To generate height response curves as a function of concentration, the response values of the last 50 s of each previous dissociation step were averaged and subtracted from the response average of the last 50 s of the relevant association step for each concentration.

BLI to assess nonspecific binding of MBP to (GST-GATE16)_2_ was conducted (Fig S5) with biotinylated AviTag-MBP (from Avidity BIS-300 to BIS-300 positive and negative control protein kit), following the above protocol.

Disruption of NCOA4^383−522^ binding to (GST-GATE16)_2_ by ATG4B was assessed by measuring BLI response using an orbital shake speed of 1,000 rpm at 30°C for a 100-s association step, with a dissociation step in BLI buffer for 150 s. ATG4B peptide (EDEDFEILSL) was preincubated with 5 μM (GST-GATE16)_2_ before measuring response at noted concentrations.

BLI for chelated and iron-bound NCOA4^383−522^ was initiated using protein prepared in a 4°C anaerobic glove box (MBraun, under 100% nitrogen gas) before immediately transferring to the Octet RED96 for assessment. The biotinylated NCOA4^383−522^ constructs were diluted to 250 nM in degassed, anaerobic BLI buffer supplemented with 5 mM DTT and loaded to a response level of 0.35 nm on the streptavidin biosensors. The loaded biosensors were immersed in 10 μM (GST-GATE16)_2_ at an orbital shake speed of 1,000 rpm at 10°C for a 100-s association step, followed by a dissociation step in anaerobic BLI buffer for 150 s. Each measurement was performed in triplicate.

### Spotted peptide array binding assay

A peptide array spanning the NCOA4^383−522^ construct consisted of 20mers offset by three amino acids that were synthesized on a cellulose membrane via SPOT synthesis by the MIT Biopolymers Laboratory. Peptides containing known GATE16 binding peptides (Pankiv et al, 2007; Olsvik et al, 2015; Skytte Rasmussen et al, 2017) derived from p62/SQSTM1 (^332^SGGDDDWTHLSS^343^), ATG4B (^384^EDEDFEILSL^393^), and FYCO1 (^1276^DDAVFDIITDEELCQIQE^1293^) were included as positive controls, and a poly-His peptide (HHHHHHGSSHHHHHHGSSHH) was added as a negative control. The membrane was briefly soaked in methanol and then washed with TBST before blocking in buffer TBSTB (TBST with 3% BSA) for 30 min at RT. The membrane was then incubated with 4 μg/ml (GST-GATE16)_2_ or GST as a negative control in TBSTB for 30 min and subjected to three washes in TBST, each lasting 3 min. Next, the bound protein was transferred to a new nitrocellulose membrane at 30 V for 1 h via wet transfer, and this membrane was blocked in TBSTB for 30 min, incubated with HRP-conjugated anti-GST antibody (RPN1236; Cytiva) at a 1:5,000 dilution, washed with TBST three times for 3 min, exposed to high-sensitivity enhanced chemiluminescent substrate (34094; Thermo Fisher Scientific), and imaged via chemiluminescence (Azure Biosystems Imager).

### Fluorescence anisotropy

Fluorescently labeled (AF Dye 488–TFP ester) and unlabeled peptides were synthesized and HPLC-purified by the MIT Biopolymers Laboratory. The peptide sequences used were as follows.

**Table:** Peptide sequences used to measure binding via fluorescence anisotropy.

Label	Sequence
ATG4B	*EDEDFEILSL
ATG9	*RDTQKLFTSSNAIHHDKDK
SFQVI	*TKAPKAMTPSRIADSFQVI
SAQVI	*TKAPKAMTPSRIADSAQVI
FAECV	*NEPCTSFAECVCDENCEKEA
AAECV	*NEPCTSAAECVCDENCEKEA
* marks location of AF Dye 488	

For competitive fluorescence anisotropy assays, 1.5 μM mGATE16 was mixed with 10 nm labeled ATG4B peptide in a buffer FA (20 mM Tris, 150 mM NaCl, pH 7.5) at RT. Increasing concentrations of the relevant unlabeled peptide were added to individual aliquots of mGATE16 complexed to the labeled ATG4B peptide and incubated for 20 min at RT. The emission for each 120 μl sample at 520 nm (excitation at 485 nm) for both horizontally (I_VH_) and vertically (I_VV_) polarized signal, along with a G_factor_, was measured on a Photon Technology International (PTI) fluorimeter in a quartz cuvette. From these measurements, an anisotropy value r was calculated by the PTI software as (I_VV_*G_factor_-I_VH_)/(I_VV_ + 2*G_factor_*I_VH_). The ATG9 peptide was used as a negative control as this should not bind to the LDS of GATE16 (Nishimura & Tooze, 2020).

For fluorescence anisotropy assays to measure affinity, 10 nM labeled ATG4B peptide was added to individual aliquots of increasing concentrations of His_6_-GATE16 in buffer FA. After the peptide was incubated with His_6_-GATE16 for 20 min at RT, the horizontally and vertically polarized fluorescence emission at 520 nm, along with a G_factor_, was measured as stated above. These same measurements were also taken for all His_6_-GATE16 concentrations as background without the labeled peptide. Parallel and perpendicular emission signal intensities for His_6_-GATE16 alone were subtracted from those of His_6_-GATE16 with labeled peptide for each concentration before calculating an anisotropy value r. A curve of anisotropy as a function of His_6_-GATE16 concentration was fit in GraphPad Prism to a binding isotherm to obtain a K_D_.

### Protein thermal stability shift assay

NCOA4^383−522^ constructs were diluted to 10 μM in buffer FA and mixed 1:1,000 (vol/vol) with SYPRO Orange (S5692; Sigma-Aldrich). Each construct was assayed in triplicate in a 384-well plate using Applied Biosystems QuantStudio 5 Real-Time PCR System. A negative control consisting of SYPRO Orange in buffer was included in all assays. Melt curves for each sample were generated by tracking the fluorescence emission signal at 570 nm (excitation at 470 nm) as the samples were heated from 25°C to 95°C in 1°C increments. Melting temperatures were calculated as the temperature corresponding to the maximum of the first derivative of the fluorescence signal.

### Size-exclusion chromatography coupled to multi-angle light scattering (SEC-MALS)

All SEC-MALS experiments were conducted using a Dawn 8 MALS with an in-line Optilab differential refractive index detector (Wyatt). For both NCOA4^383−522^ and (GST-GATE16)_2_, 100 μl of 1 mg/ml protein was injected onto an equilibrated WTC-030 HPLC SEC column in buffer FA at a flow rate of 0.5 ml/min. The instrument was calibrated with a 2 mg/ml BSA standard before each run. Data were analyzed with Astra software (Wyatt) to report apparent molecular weight.

### Western blot assays of ferritinophagy in vivo

Stably integrated NCOA4^383−522^ cell lines were generated via retroviral transfection of the relevant NCOA4^383−522^ construct in a CMV-enhancer–bearing pMRX plasmid (84573; Addgene) into NCOA4Δ HeLa cells (a gift from Prof. M. Poli, University of Brescia, Italy) (Gryzik et al, 2021). In this pMRX plasmid, EGFP was fused to the N terminus of NCOA4^383−522^ and its mutants with a 13-residue GS linker. HeLa cells were transfected and selected as described previously (Sena-Esteves & Gao, 2018).

WT HeLa (cl_JD066), NCOA4Δ (cl_JD065), NCOA4^383−522^ (cl_JD074), ^413^F:A (cl_JD075), and ^486^F:A (cl_JD076) cell lines were seeded in a 12-well cell culture plate in DMEM (Genesee Scientific) and incubated at 37°C, 5% CO_2_. After 24-h, all media were aspirated and replaced with either DMEM or DMEM supplemented with 40 μM ferric ammonium citrate (FAC). After an additional 24 h, all media were aspirated and replaced with either DMEM or DMEM supplemented with 50 μM deferoxamine (DFO). After an additional 24-h incubation, cells were trypsinized (200 μl, 5 min, 37°C), pelleted via centrifugation at 100*g* for 1 min, resuspended in Dulbecco’s PBS (D8537; Sigma-Aldrich), and pelleted as above before undergoing flash freezing in liquid nitrogen and storage at −80°C.

Frozen cell pellets were resuspended in 100 μl 4X Laemmli buffer with Halt protease and phosphatase inhibitor (78440; Thermo Fisher Scientific) and lysed with a 29-gauge syringe. Samples were mixed with 285 mM dithiothreitol, boiled, and run on an SDS–PAGE gel before being transferred to a PVDF membrane (Invitrogen) using an iBlot2 transfer device (7 min; 20 V; Invitrogen). Membranes were blocked overnight in TBSTB, and incubated with rabbit anti-FTH1 antibody diluted 1:1,000 (3998S; Cell Signaling) or HRP-conjugated anti-tubulin antibody diluted 1:20,000 (GTX628802-01; GeneTex) as above. Anti-rabbit HRP-conjugated secondary antibody (AS014; ABclonal) was applied at a 1:1,000 dilution and washed, exposed to enhanced chemiluminescent substrate, and imaged as above. Ferritin levels were then measured by densitometry in FIJI (Schindelin et al, 2012) and normalized to tubulin intensity measured as above.

### Fluorescence assays

For colocalization assays, NCOA4^383−522^ cell lines were seeded on glass coverslips in a 12-well cell culture plate in DMEM as above. After 24 h, media were aspirated and replaced with DMEM supplemented with 50 μM DFO, and cells were incubated for an additional 24 h. Cells were then fixed with 4% paraformaldehyde in PBS (150 mM NaCl, 2.7 mM KCl, 10 mM Na_2_HPO_4_, 1.8 mM KH_2_PO_4_, pH 8.0) for 15 min at RT, quenched with 100 mM glycine in PBS for 10 min, and washed three times with PBS. Cells were next permeabilized with 0.5% Triton X-100 in PBS for 10 min, washed once with PBS, and blocked for 30 min in 2% BSA in PBS. Slides were next incubated in anti-GFP mouse antibody (sc-9996; Santa Cruz) diluted 1:200, anti-LAMP1 rabbit antibody (9091T; Cell Signaling) diluted 1:200, and Hoechst stain (H3570; Invitrogen) diluted 1:1,000, in 2% BSA in PBS for 1 h. After three 5-min soaks in PBS, slides were incubated in Alexa Fluor 488 anti-mouse antibody (A-11001; Thermo Fisher Scientific) diluted 1:200, and Alexa Fluor 568 anti-rabbit antibody (A-11036; Thermo Fisher Scientific) diluted 1:200, for 1 h. Slides were then soaked three times for 5-min in PBS, dried and mounted on slides with ProLong Gold mounting medium, set overnight, and sealed. Slides were imaged on a Dragonfly 505 spinning-disk confocal microscope with an iXon Ultra 888 EMCCD camera with the following settings: 60x objective pixel size (206 μm × 206 μm), 40 μm pinhole size, 0.5-µm interval z-stack slices, 405-nm laser with 445/46 band-pass emission filter, 488-nm laser with 521/38 band-pass filter, and 561-nm laser with 594/43 band-pass filter. A minimum of 100 cells were imaged per condition.

All image analysis was conducted in FIJI (Schindelin et al, 2012). To measure the area of overlap per cell, the best focused image from the z-stack was first manually chosen based on the nuclei in the 405-nm channel. The in-focus image was then split into its respective green (NCOA4^383−522^) and pink (lysosome) channels before merging into a single RGB image. Images were viewed at fixed brightness and saturation when selecting regions of overlap (white). The overlapping regions were then measured, and the area per nucleus was recorded. The JACoP plugin for FIJI (Bolte & Cordelières, 2006) was used to estimate the fraction of lysosomes that colocalized with NCOA4^383−522^. In brief, each z-stack was split into the respective channels, background-subtracted with a rolling ball radius of 50 pixels, and set to standard brightness values. For all images, threshold values were manually chosen for the pink and green channels, and Manders’ value, which calculates the fraction of the pink signal that overlaps green signal, was recorded.

To monitor autophagic punctum formation, we evaluated the specificity of commercially available antibodies against LC3B (EPR18709; Abcam) and GATE16 (ab137511; Abcam; PA5-116443; Thermo Fisher Scientific; abx108277; Abbexa) by Western blot analysis of WT HeLa cells and HeLa cells lacking all six Atg8-family proteins (Nguyen et al, 2016). Only the LC3B antibody exhibited the expected loss of signal in knockout cells, whereas all tested GATE16 antibodies produced similar banding patterns in WT and knockout lysates, precluding their use for specific detection. Thus, we probed for these puncta using this reagent. Briefly, WT HeLa or ATG7Δ HeLa cells (Mejlvang et al, 2018) were seeded on glass coverslips in a 12-well cell culture plate in DMEM. After 24 h, media were aspirated and replaced with DMEM supplemented with 40 μM FAC or regular DMEM, and cells were incubated for an additional 24 h. Media were then aspirated and replaced with DMEM supplemented with 50 μM DFO with or without 400 nM BafA or regular DMEM, and cells were incubated for an additional 24 h. Cells were then fixed and stained according to the above protocol using anti-LC3B primary antibody (EPR18709; Abcam) and Alexa Fluor 568 anti-rabbit secondary antibody (A-11036; Thermo Fisher Scientific). Slides were imaged with widefield fluorescence with a 50-ms exposure on a Nikon Eclipse Ti2 inverted microscope using a 60x oil immersion objective, and the C-FL DSRed filter set (554-nm excitation, 609-nm emission).

### NCOA4^383−522^ iron chelation and reconstitution

To obtain the reconstituted NCOA4^383−522^ sample, purified NCOA4^383−522^ was diluted twofold in buffer IC (20 mM Tris, 150 mM NaCl, 5 mM DTT, pH 7.5, degassed and stored anaerobically) incubated with a 10-fold molar excess of ammonium iron (II) sulfate hexahydrate and sodium sulfide nonahydrate overnight at 20°C in an anaerobic glove box. Excess iron and sulfur were pelleted via centrifugation at 17,200*g* for 2 mins and filtered using a centrifuge tube 0.22-μm filter (8160; Corning) before loading onto an S200 10/300 Increase size-exclusion column run in buffer IC under anaerobic conditions. Fractions eluting at ∼0.57 column volumes were collected and concentrated with 30-kD Centricon for use in BLI.

To obtain the chelated sample, purified NCOA4^383−522^ was incubated with EDTA and potassium ferricyanide in molar ratios of 1:50 and 1:20, respectively, for 30 min at RT. This material was run over an S200 10/300 Increase in buffer IC. Fractions eluting at ∼0.57 column volumes were collected and concentrated for subsequent use in BLI.

Chelated and reconstituted NCOA4^383−522^ were both tested in a ferene assay (Fish, 1988; McCarthy & Booker, 2018; Levitz et al, 2022) to measure iron concentration. In brief, chelated and reconstituted NCOA4^383−522^ samples were diluted to 2.5 μM in buffer IC and iron (III) nitrate nonahydrate (047282.AP; Thermo Fisher Scientific) standards spanning 0–100 μM were made in volumes of 100 μl. Samples and standards were mixed with 100 μl of Reagent A (156 mM SDS, 113 mM saturated sodium acetate) and 100 μl of Reagent B (274 mM ascorbic acid, 8 mM sodium meta-bisulfite, 536 mM saturated sodium acetate) and then incubated at 30°C for 15 min. 5 μl of Reagent C (36 mM ferene) was added before centrifuging samples at 21,130*g* for 5 min. The absorbance at 592 nm for 250 μl of supernatant from each condition was then measured in a 96-well clear plate using a Molecular Devices plate reader. Three replicates of each protein sample were compared with a standard curve calculated from the standards to obtain an iron concentration.

## Supplementary Material

Reviewer comments

## Data Availability

All data supporting the work are in the article itself or will be shared upon reasonable request.
