# Supplementary material for: NCOA4 initiates ferritinophagy by avidly binding GATE16 using two short linear interaction motifs
Source: Life Sci Alliance. 2026 Feb 4;9(4):e202503611. doi: 10.26508/lsa.202503611 (PMC12872393; doi:10.26508/lsa.202503611)
Supplement: Reviewer comments [file LSA-2025-03611_review_history.pdf]

NCOA4 initiates ferritinophagy by avidly binding GATE16 using two short linear interaction motifs

April Lee and Joseph Davis
DOI: https://doi.org/10.26508/lsa.202503611

Corresponding author(s): Joseph Davis, Massachusetts Institute of Technology

**Review Timeline:**		
	Submission Date:	2025-12-22
	Editorial Decision:	2026-01-07
	Revision Received:	2026-01-16
	Accepted:	2026-01-20

Scientific Editor: Tim Fessenden

Transaction Report:

(Note: With the exception of the correction of typographical or spelling errors that could be a source of ambiguity, letters and reports are not edited. The original formatting of letters and referee reports may not be reflected in this compilation.)

No full Peer Review Process File is available with this article, as the authors have chosen not to make the review process public in this case.

Please note that the manuscript was previously reviewed at another journal and the reports were taken into account in the decision-making process at *Life Science Alliance*.

January 7, 2026

RE: Life Science Alliance Manuscript #LSA-2025-03611-T

Prof. Joseph Davis
Massachusetts Institute of Technology
Biology
77 Massachusetts Ave.
Cambridge, MA 02139

Dear Dr. Davis,

Thank you for transferring your revised manuscript entitled "NCOA4 initiates ferritinophagy by avidly binding GATE16 using two short linear interaction motifs" to Life Science Alliance. In view of these important findings and the satisfactory resolution of major reviewer concerns, we will be happy to publish your paper in Life Science Alliance pending a few final revisions.

Please also tend to the following as well as additional formatting requirements below:

-Please upload all figure files individually, including the supplementary figure files; all figure legends should only appear in the main manuscript file.
-Please remove legends from the supplementary figures.
-Please add a Category and Keywords for your manuscript in our system
-Please add a Summary Blurb/Alternate Abstract in our system.
-Please add the X and Bluesky handles of your host institute/organization, as well as your own and/or one of the authors, in our system.
-Please be sure that the authorship listing and order are correct and match between the system and the manuscript file.
-Please add authors' contributions to our system as well.
-Please add a Conflict of Interest statement to your main manuscript text.
-Please include a "Data Availability" section after the Materials & Methods section. Please consult our guidelines at https://www.life-science-alliance.org/manuscript-prep#format
-Please move your main and supplementary figure legends in the main manuscript text after the references section.
-Please add callout for Figures 2A-B; S1A-C; S2A-C; S3A-B; S6A-B; S7A-B; S8A-B; S11A-B and S12A-B to your main manuscript text.
-Please add molecular weight markers to the blots in Figure S11A.

If you are planning a press release on your work, please inform us immediately to allow informing our production team and scheduling a release date.

LSA now encourages authors to provide a 30-60 second video where the study is briefly explained. We will use these videos on social media to promote the published paper and the presenting author (for examples, see https://docs.google.com/document/d/1-UWCfbE4pGcDdcgzcmiuJl2XMBJnxKYeqRvLLrLSo8s/edit?usp=sharing). Corresponding or first-authors are welcome to submit the video. Please submit only one video per manuscript. The video can be emailed to contact@life-science-alliance.org

To upload the final version of your manuscript, please log in to your account: https://lsa.msubmit.net/cgi-bin/main.plex
You will be guided to complete the submission of your revised manuscript and to fill in all necessary information. Please get in touch in case you do not know or remember your login name.

To avoid unnecessary delays in the acceptance and publication of your paper, please read the following information carefully.

A. FINAL FILES:

These items are required for acceptance.

-- An editable version of the final text (.DOC or .DOCX) is needed for copyediting (no PDFs).

-- High-resolution figure, supplementary figure and video files uploaded as individual files: See our detailed guidelines for preparing your production-ready images, https://www.life-science-alliance.org/authors

-- Summary blurb (enter in submission system): A short text summarizing in a single sentence the study (max. 200 characters including spaces). This text is used in conjunction with the titles of papers, hence should be informative and complementary to the title. It should describe the context and significance of the findings for a general readership; it should be written in the present tense and refer to the work in the third person. Author names should not be mentioned.

B. MANUSCRIPT ORGANIZATION AND FORMATTING:

Full guidelines are available on our Instructions for Authors page, https://www.life-science-alliance.org/authors

We encourage our authors to provide original source data, particularly uncropped/-processed electrophoretic blots and spreadsheets for the main figures of the manuscript. If you would like to add source data, we would welcome one PDF/Excel-file per figure for this information. These files will be linked as supplementary "Source Data" files.

Submission of a paper that does not conform to Life Science Alliance guidelines will delay the acceptance of your manuscript.

It is Life Science Alliance policy that if requested, original data images must be made available to the editors. Failure to provide original images upon request will result in unavoidable delays in publication. Please ensure that you have access to all original data images prior to final submission.

The license to publish form must be signed before your manuscript can be sent to production. A link to the license to publish form will be available to the corresponding author only. Please take a moment to check your funder requirements.

Reviews, decision letters, and point-by-point responses associated with peer-review at Life Science Alliance will be published, alongside the manuscript. If you do want to opt out of having the reviewer reports and your point-by-point responses displayed, please let us know immediately.

Thank you for your attention to these final processing requirements. Please revise and format the manuscript and upload materials as soon as you are able.

Thank you for this interesting contribution, we look forward to publishing your paper in Life Science Alliance.

Sincerely,

Tim Fessenden
Scientific Editor
Life Science Alliance

January 20, 2026

RE: Life Science Alliance Manuscript #LSA-2025-03611-TR

Prof. Joseph H Davis
Massachusetts Institute of Technology
Biology
77 Massachusetts Ave.
Cambridge, MA 02139

Dear Dr. Davis,

Thank you for submitting your Research Article entitled "NCOA4 initiates ferritinophagy by avidly binding GATE16 using two short linear interaction motifs". It is a pleasure to let you know that your manuscript is now accepted for publication in Life Science Alliance. Congratulations on this interesting work.

The final published version of your manuscript will be deposited by us to PubMed Central upon publication.

Your manuscript will now progress through copyediting and proofing. It is journal policy that authors provide original data upon request.

Reviews, decision letters, and point-by-point responses associated with peer-review at Life Science Alliance will be published, alongside the manuscript. If you want to opt out of having the reviewer reports and your point-by-point responses displayed, please let us know immediately.

IMPORTANT: If you will be unreachable at any time, please provide us with the email address of an alternate author. Failure to respond to routine queries may lead to unavoidable delays in publication.

Scheduling details will be available from our production department. You will receive proofs shortly before the publication date. Only essential corrections can be made at the proof stage so if there are any minor final changes you wish to make to the manuscript, please let the journal office know now.

DISTRIBUTION OF MATERIALS:
Authors are required to distribute freely any materials used in experiments published in Life Science Alliance. Authors are encouraged to deposit materials used in their studies to the appropriate repositories for distribution to researchers.

You can contact the journal office with any questions, contact@life-science-alliance.org

Again, congratulations on a very nice paper. I hope you found the review process to be constructive and are pleased with how the manuscript was handled editorially. We look forward to future exciting submissions from your lab.

Sincerely,

Tim Fessenden
Scientific Editor
Life Science Alliance
